# Salinomycin co-treatment enhances tamoxifen cytotoxicity in luminal A breast tumor cells by facilitating lysosomal degradation of receptor tyrosine kinases

**DOI:** 10.18632/oncotarget.10459

**Published:** 2016-07-07

**Authors:** Ann-Katrin Sommer, Adam Hermawan, Frauke Martina Mickler, Bojan Ljepoja, Pjotr Knyazev, Christoph Bräuchle, Axel Ullrich, Ernst Wagner, Andreas Roidl

**Affiliations:** ^1^ Pharmaceutical Biotechnology, Department of Pharmacy, Ludwig-Maximilians-Universität München, 81377 Munich, Germany; ^2^ Department of Molecular Biology, Max-Planck-Institute of Biochemistry, 82152 Martinsried, Germany; ^3^ Physical Chemistry, Department of Chemistry, Ludwig-Maximilians-Universität München, 81377 Munich, Germany

**Keywords:** tamoxifen, resistance, salinomycin, endosomal trafficking, breast cancer

## Abstract

Luminal A breast cancer is the most common breast cancer subtype which is usually treated with selective estrogen receptor modulators (SERMS) like tamoxifen. Nevertheless, one third of estrogen receptor positive breast cancer patients initially do not respond to endocrine therapy and about 40% of luminal A breast tumors recur in five years. In this study, we investigated an alternative treatment approach by combining tamoxifen and salinomycin in luminal A breast cancer cell lines. We have found that salinomycin induces an additional cytotoxic effect by inhibiting the ligand independent activation of ERα. Thereby salinomycin increases the intracellular calcium level. This leads to a premature fusion of endosomes with lysosomes and thus to the degradation of Egfr family members. Since this process is essential for luminal A breast cancer cells to circumvent tamoxifen treatment, the combination of both drugs induces cytotoxicity in tamoxifen sensitive as well as resistant luminal A breast cancer cell lines.

## INTRODUCTION

According to the American Cancer Society breast cancer is still the second leading cause of cancer deaths among women in which luminal A is the most common subtype (70 %) [1, 2]. It is characterized by high estrogen receptor α (ERα) as well as progesterone receptor expression levels [3, 4]. Consequently, endocrine therapy with selective estrogen receptor modulators (SERMs) like tamoxifen is the first line treatment for this type of breast cancer. This drug class acts as agonist-antagonist, as it mediates an agonistic effect on ERα, e.g. in bone tissue and thus prevents women from osteoporosis. In parallel, it works as an antagonist on ERα in breast cancer tissue where it inhibits tumor growth in estrogen dependent breast cancer [5]. However, acquired or intrinsic resistance occurs in about 40% of breast cancer patients due to three major resistance mechanisms [6]: Overexpression of multidrug resistance protein 1 (MDR-1), the occurrence of so called cancer stem cells (CSCs) as well as especially in this breast cancer subtype the ligand independent activation of ERα via different Receptor Tyrosine Kinases (RTKs) [7–9]. For that reason, additional therapy options need to be investigated to improve patient outcome. It was previously shown that the carboxylic polyether antibiotic and ionophore salinomycin is able to overcome MDR-1 as it integrates in biological membranes [10]. Moreover, CSCs can be eradicated by salinomycin [11, 12]. Accordingly, it was recently determined that this antibiotic circumvents apoptosis-resistance by inhibition of autophagy depending on the available glucose level [13–16].

Another important effect on cancer is that salinomycin is able to reduce metastasis formation [17–19] and inhibits EMT by activation of FOXO3a [20]. Salinomycin was also utilized as additive in several other combinational treatment approaches overcoming acquired drug resistance [20, 21].

Therefore, we investigated whether salinomycin can be utilized for a combinational approach with tamoxifen and is suitable to circumvent tamoxifen resistance. We demonstrate that salinomycin hampers the ligand independent activation of ERα which is mediated by RTKs. By increasing the intracellular calcium concentration this antibiotic facilitates Egfr and Her2 degradation via lysosomes thus blocking mitogenic signaling. Hence, salinomycin is also able to overcome tamoxifen resistance in luminal A breast cancer rendering salinomycin a promising additive anti-cancer drug.

## RESULTS

### Combinatorial treatment with tamoxifen and salinomycin shows increased cytotoxicity in luminal A breast cancer cells

**Figure 1:**
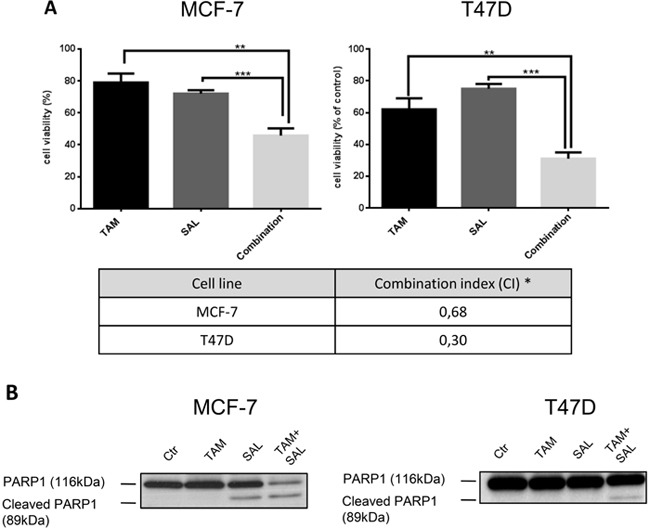
Cytotoxicity of combined treatment with tamoxifen and salinomycin **A.** Cytotoxicity assay of treated MCF-7 and T47D cells. Cell viability was assessed upon 72h treatment with 10μM tamoxifen, 0,5μM salinomycin or the combination using Cell Titer Glo® reagent. N=6; * CI<1: synergistic, CI=1: additive, CI>1 antagonistic. **B.** PARP1 cleavage. Western blot analysis of two luminal A breast cancer cell lines MCF-7 and T47D upon 72h treatment with 10μM tamoxifen, 0,5μM salinomycin or their combination. Total cell lysates were analyzed for PARP1 cleavage, an apoptosis marker. p-values: *0,05; **0,01; ***0,001; ****0,0001.

To investigate whether salinomycin has a beneficial effect on the therapy of luminal A breast cancer, it was used as single drug or in combination with tamoxifen to treat MCF-7 and T47D luminal A breast cancer cell lines. Cell viability was examined after 72h of treatment. By calculating the combination index, we observed in both cell lines a synergistic effect of the combinational treatment with tamoxifen and salinomycin [22–24] (Figure 1A). To further validate the increased cytotoxicity poly ADP ribose polymerase (PARP) cleavage, a biomarker for cell death, was analyzed by western blotting. Here, PARP1 was cleaved upon salinomycin treatment in MCF-7 and the combinatorial treatment with salinomycin and tamoxifen in both cell lines (Figure 1B).

### Salinomycin inhibits the ligand independent activation of ERα

The underlying mechanism of the additional cytotoxic effect of salinomycin needs to be further elucidated because MCF-7 and T47D show no overexpression of ABC transporters as well as cancer stem cell-like properties. Therefore, we investigated the important signaling pathways in luminal A breast cancer cells by western blotting upon 72h treatment with tamoxifen, salinomycin or the combination.

**Figure 2:**
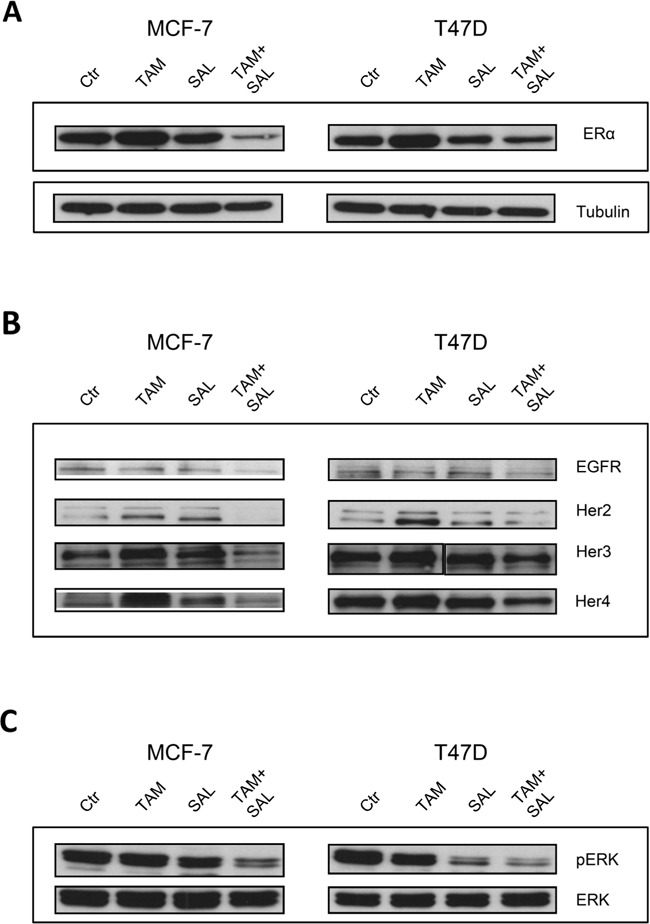
Characterization of cytotoxicity Western blot analysis of two luminal A breast cancer cell lines MCF-7 and T47D upon 72h treatment with 10μM tamoxifen, 0,5μM salinomycin or the combination. Tubulin was used as loading control and is representative for all blots. **A.** Expression of ERα upon treatment. To investigate the protein expression of ERα total cell lysates were utilized. **B.** Ligand independent signaling of ERα. Total cell lysates were analyzed for protein expression levels of Egfr-family members. **C.** Ligand independent signaling of Erk1/2. To investigate the protein expression of Erk1/2 total cell lysates were utilized.

Tamoxifen treatment increases the protein expression of ERα reflecting a common mechanism to circumvent target inhibition. Salinomycin treatment on the other hand is not changing the expression level of ERα but importantly the combination is decreasing the amount of ERα compared to control (Figure 2A).

Another important pathway in hormone receptor positive breast cancer is the ligand independent activation of ERα by Egfr-family members which represents an intrinsic resistance mechanism to endocrine therapy. For that reason, the protein expression of Egfr, Her2, Her3 and Her4 were analyzed by western blotting. We found that single tamoxifen treatment increases the expression levels of Her2 (in both cell lines) and Her3 as well as Her4 in MCF-7 cells compared to untreated cells. No changes were detectable in the protein expression of Egfr-family members upon salinomycin treatment. Noteworthy, by applying the combination of tamoxifen and salinomycin the expression level of all Egfr-family members was reduced. We additionally analyzed the gene expression by qPCR and observed a slight reduction after 72 h in all treatment conditions (Supplementary Figure S1). As a consequence of the reduced RTK expression downstream Erk phosphorylation was diminished, too (Figure 2B and 2C).

These results demonstrate that a combination of tamoxifen with salinomycin decreased the expression of RTKs and efficiently inhibits the ligand independent signaling which is usually turned on after single tamoxifen treatment. By combining tamoxifen with salinomycin it is possible to circumvent one major tamoxifen resistance mechanism.

### Salinomycin facilitates the lysosomal degradation of RTKs

**Figure 3:**
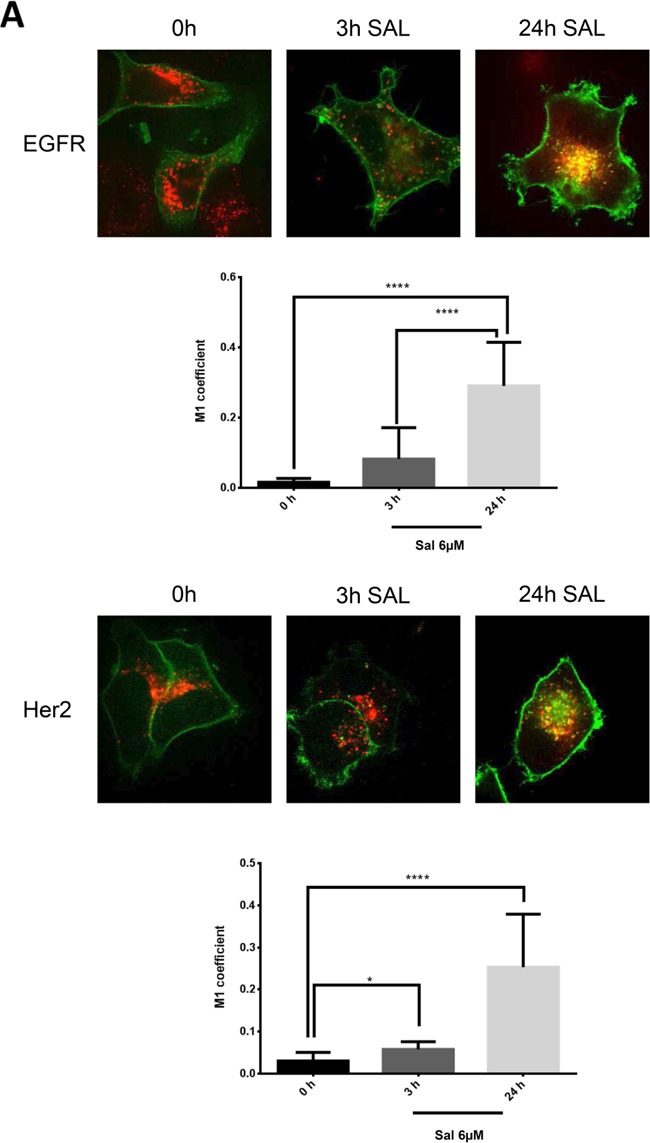

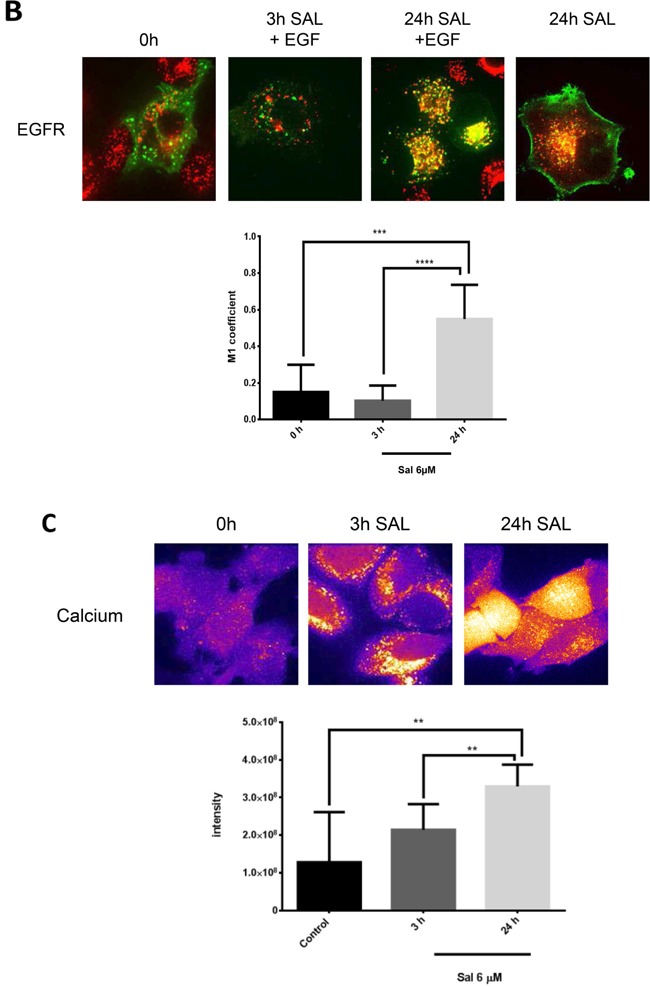

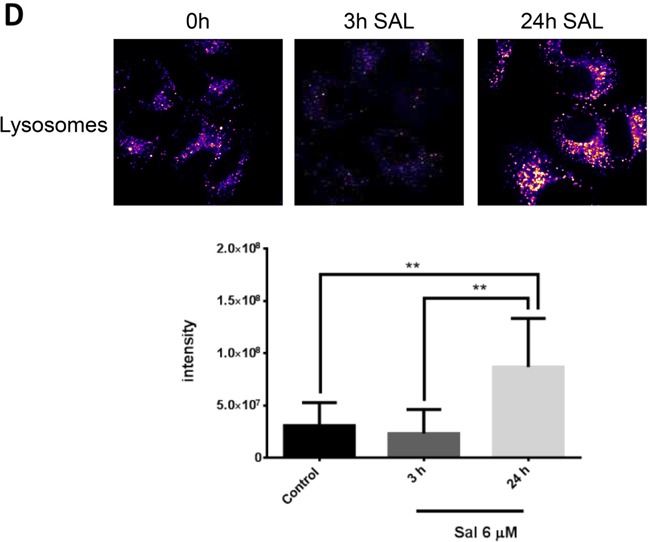
Endocytosis of Egfr-family members is altered upon salinomycin treatment **A.** Egfr and Her2 accumulate in lysosomes MCF-7 cells were transfected with Egfr-eGFP or Her2-eGFP plasmid. Receptor-GFP expressing cells were imaged 48-72 hours after transfection by spinning disk microscopy. To detect the effect of salinomcyin on receptor trafficking, salinomycin was added at 6μM concentration 3h and 24h before imaging. For co-localization with lysosomes, 15nM lysotracker deep red was added. Co-localization was quantified by Image J JaCOP plug-in and displayed as Manders coefficient M1 (N=7-13 images per incubation time). Image size 62μm. **B.** Salinomycin inhibits receptor recycling upon EGF-stimulation MCF-7 cells were transfected with Egfr-eGFP plasmid. Receptor-GFP expressing cells were imaged 48-72 hours after transfection by spinning disk microscopy. To detect the effect of salinomcyin on receptor trafficking, salinomycin was added at 6μM concentration 3h and 24h before imaging. For co-localization with lysosomes, 15nM lysotracker deep red was added. To induce receptor endocytosis, 50 pmol/ml EGF was added to the cells. Co-localization was quantified by Image J JaCOP plug-in and displayed as Manders coefficient M1. (N=7-13 images per incubation time). Image size 62μm. **C.** Ca^2+^-levels are elevated MCF-7 cells were pretreated with 6μM salinomycin for 3h, 24h or kept without salinomycin treatment. 1 hour before imaging, 6μM Fluo-3-AM was added to the cells. To quantify the Fluo-3-AM fluorescence in the cells, digital image analysis was performed in ImageJ. Mean values of all evaluated cells are presented (N=8-10 images for each incubation time). **D.** Measurement of the intensity of lysosomes. MCF-7 cells were pretreated for 3h, 24h with 6μM salinomycin or kept without salinomycin treatment. 1 hour before imaging 15nM Lysotracker was added to the cells. Digital image analysis was utilized in ImageJ to quantify Lysotracker fluorescence in the cells. Mean values of all evaluated cells are presented (N=8-10 images for each incubation time) Image size 62μm. p-values: *0,05; **0,01; ***0,001; ****0,0001.

To elucidate the mechanism how the ionophore salinomycin is able to interfere with the ligand independent activation of ERα via Egfr-family members we analyzed the cellular distribution of Egfr and Her2 during salinomycin treatment with spinning disk microscopy. We transfected Egfr and Her2 with a GFP-tag into MCF-7 breast cancer cells to monitor their cellular fate upon salinomycin treatment. Interestingly, we found that salinomycin induces a strong co-localization of these RTKs with the lysosomal marker lysotracker suggesting an enhanced lysosomal degradation after 24h of salinomycin treatment. This is further supported by the quantification of the co-localization with the M1-coefficient (Figure 3A). When cells were stimulated with EGF and treated with salinomycin the receptor disappeared from the membrane and almost completely accumulated in lysosomes after addition of salinomycin. In contrast, without EGF stimulation cells still displayed a cell surface staining indicating a balance between lysosomal degradation and *de novo* synthesis of these RTKs (Figure 3B).

Salinomycin is able to increase the intracellular calcium level as previously shown in neurons [25]. This elevated calcium level, among other factors, is responsible for enhanced endocytosis and premature fusion of lysosomes with endosomes [26, 27]. Thus, we wanted to elucidate whether salinomycin treatment also augments the cytosolic calcium level in breast cancer cells. Our results demonstrate that the intracellular calcium level is significantly increased in MCF-7 cells upon salinomycin treatment as observed by a Fluo-3-AM staining (Figure 3C). By applying lysotracker we found an increased number of lysosomes accordingly.

They also seem to be stalled and are no longer transported within the cytoplasm, as depicted in a time projection and videos by live cell imaging of salinomycin-treated HeLa cells (Figure 3D and Supplementary Figure S2). Taken together, these results show that salinomycin reduces the overall protein amount of the Egfr-family by enhanced lysosomal degradation. Furthermore, the salinomycin-triggered elevated cytosolic calcium level leads to pre-mature fusion of endosomes with lysosomes and accounts for decreased RTK expression.

### Sequential tamoxifen treatment of MCF7 cells mimics luminal A breast cancer resistance

Tamoxifen and other SERMS are considered as first-line treatment of ERα-positive breast cancer. Nevertheless, resistance to endocrine therapy occurs in about 40% of these breast cancer patients causing tumor relapse. As salinomycin displayed beneficial properties when combined with tamoxifen, we generated a tamoxifen resistant model cell line to further investigate the efficacy of the combination.

**Figure 4:**
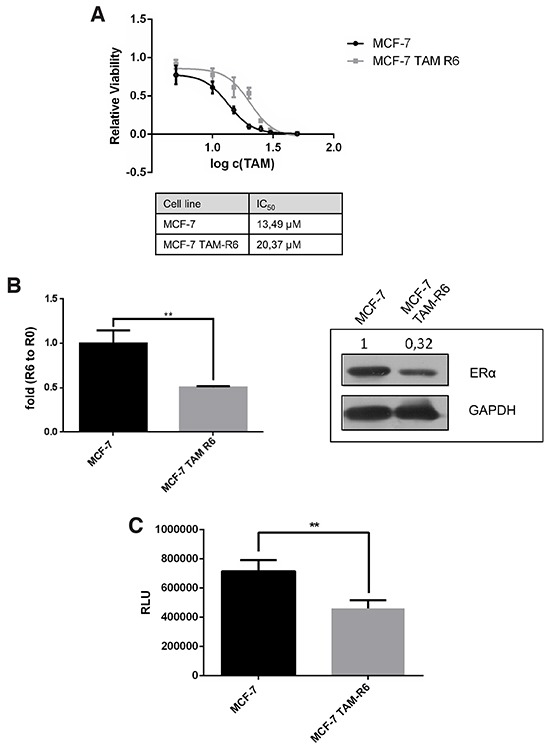

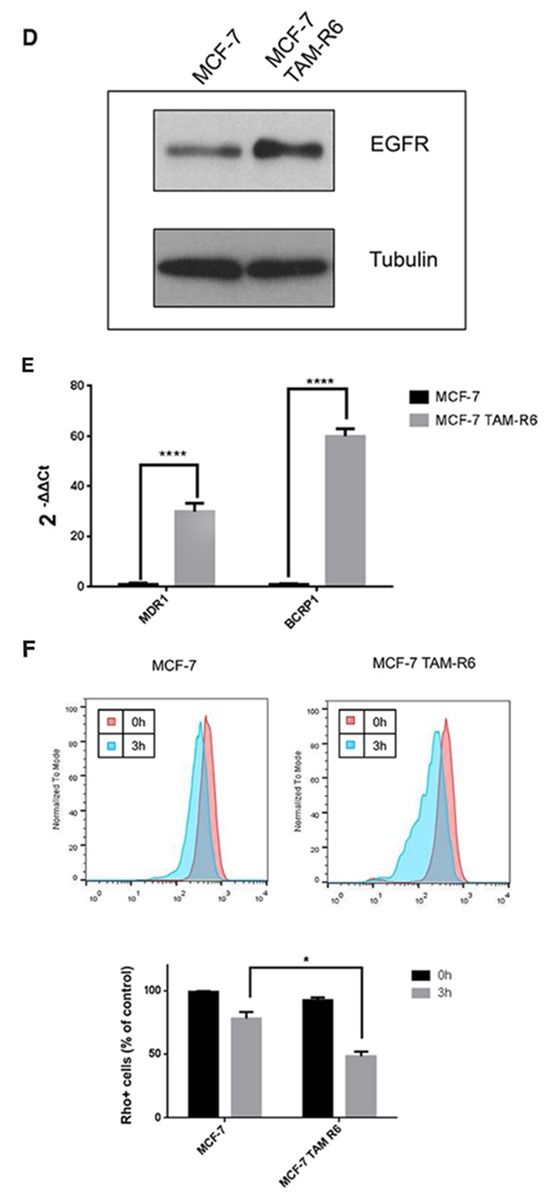

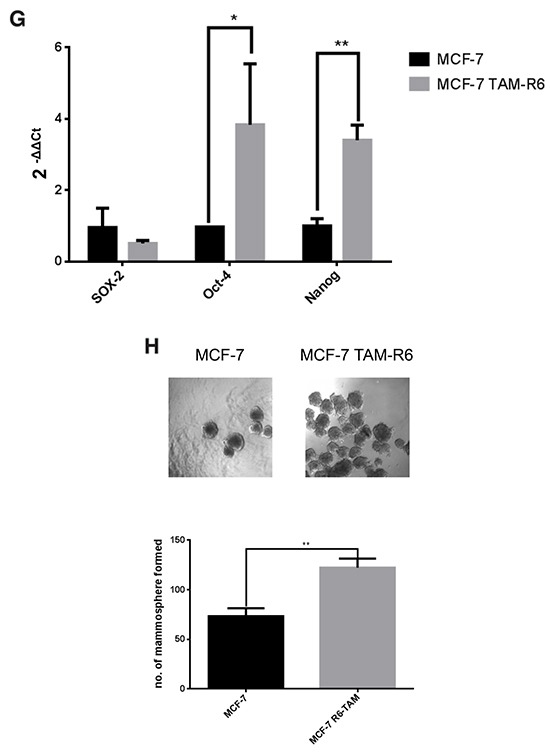
Tamoxifen resistance MCF-7 cells were treated in several rounds with 10μM tamoxifen for 72h followed by recovery phase until a confluence of 80%. Cells that received either no treatment (MCF-7) or six treatment rounds (MCF-7 TAM-R6) were harvested for the following experiments. **A.** IC_50_ of tamoxifen in resistant cells is increased. Cytotoxicity assay of tamoxifen resistant MCF-7 cells. MCF-7 TAM-R6 cells were treated with indicated concentrations of tamoxifen for 72h. Subsequently cell viability was assessed by Cell Titer Glo® Assay (Promega). N=3. **B.** ERα expression in MCF-7 TAM-R6 is reduced. Quantitative RT-PCR analysis of ERα level in MCF-7 vs. MCF-7 TAM-R6 cells. Expression levels were normalized to parental MCF-7 cells. N=3 Protein expression of ERα in parental MCF-7 as well as MCF-7 TAM-R6 were analyzed by western blotting. **C.** Analysis of ERα signaling Parental MCF-7 and MCF-7 TAM-R6 cells were transfected with 3x-ERE-TATA-Luc plasmid (Addgene #11354) and serum-starved for 12h. Next, cells were stimulated with 1nM estradiol for 30 min and subsequently treated with 10μM tamoxifen for 12h. The luciferase signal was analyzed and normalized to control. p-values: * 0,05; ** 0,01; ***0,001; **** 0,0001. **D.** Egfr expression in MCF-7 TAM-R6 is increased. Western blot analysis of parental MCF-7 and MCF-7 TAM-R6 total cell lysates was performed to investigate the expression level of Egfr. Tubulin was used as loading control. **E.** Expression of ABC transporters is enhanced in tamoxifen resistant cells qPCR was utilized to examine the mRNA expression level of multiple-drug-resistance protein 1 (MDR1) and breast cancer resistance protein 1 (BCRP1), two ABC-transporters that are frequently causing drug resistance. **F.** Rhodamine123 efflux assay. The dye Rhodamine123 is a well-known target of ABC-transporters. Therefore, parental MCF-7 as well as MCF-7 TAM-R6 cells were treated for 30 minutes with 0,2 μg/ml Rhodamine123. Afterwards cells were washed to eliminate Rhodamine123, resuspended in the corresponding medium. Finally, flow cytometry was performed 1h, 2h and 3h later to determine the efflux activity. Data were normalized to untreated samples. **G.** Expression of cancer stem cell marker is enhanced in MCF-7 TAM-R6 compared to parental cells. To determine the occurrence of cancer stem cells in tamoxifen resistant cells three prominent CSC marker SOX-2, Oct-4 and Nanog were investigated by qPCR. **H.** Mammosphere formation is increased in tamoxifen resistant cells. Another possibility to analyze stem cell properties in cancer cell populations is the mammosphere forming assay. Therefore, 10 × 10^5^ c/w parental MCF-7 as well as MCF-7 TAM-R6 were seeded in a non-adherent 96-well plate and formed spheroids were counted 7 days afterwards.

We repeatedly treated MCF-7 breast cancer cells with tamoxifen (these cells are termed MCF-7 TAM-R6 in the following). After six treatment cycles the sensitivity to tamoxifen was determined. In MCF-7 TAM-R6 cells the IC_50_ was 1,5x higher compared to parental MCF-7 cells reflecting acquired tamoxifen resistance (Figure 4A). Moreover, we observed a decreased mRNA level as well as protein amount of estrogen receptor alpha (ERα) in MCF-7 TAM-R6 cells (Figure 4B).

As tamoxifen binds to ERα one reason for resistance to endocrine therapy is the loss of its target. We further analyzed the ERα pathway in MCF-7 TAM-R6 by utilizing a luciferase reporter plasmid with a promotor, containing estrogen responsive elements, to monitor ERα-mediated gene transcription. By comparing parental MCF-7 cells with tamoxifen resistant cells, we observed that the ERα signaling is significantly diminished in MCF-7 TAM-R6 cells (Figure 4C). Additionally the expression level of Egfr in parental MCF-7 and MCF-7 TAM-R6 was investigated. Since the protein amount of Egfr was increased in tamoxifen resistant cells, the ligand independent activation of ERα via Egfr in MCF-7 TAM-R6 cells plays an even more important role than in parental MCF-7 cells (Figure 4D).

Besides ligand independent activation, tamoxifen resistance can be implemented by a variety of further molecular resistance mechanisms. Amongst them are the increase of efflux pumps as well as the appearance of so called cancer stem cells (CSC). To further characterize MCF-7 TAM-R6 cells the mRNA levels of two ABC-transporters, multidrug resistance protein 1 (MDR1) and breast cancer resistance protein (BCRP1), were examined.

We observed that repeated tamoxifen treatment augments the expression of both multidrug resistance pumps causing an increased efflux of drugs and thus acquired resistance to tamoxifen (Figure 4E). In order to investigate the functionality of these ABC-transporters an efflux assay with rhodamine was performed. We could show that the efflux of rhodamine after 3h was increased in tamoxifen resistant cells compared to parental MCF-7 (Figure 4F).

By investigating the expression level of the three crucial stem cell markers Sox2, Nanog and Oct4, we want to provide evidence for the existence of cancer stem cell-like traits in tamoxifen resistant cells, which are known to generate drug resistance. Six treatment cycles of tamoxifen increased the mRNA levels of Nanog and Oct4 in MCF-7 TAM-R6 significantly, whereas no change in the Sox2 expression was observed (Figure 4G).

To functionally prove the existence of cancer stem cells, we performed a mammosphere forming assay and determined an increased amount of formed spheroids in MCF-7 TAM-R6 cells (Figure 4H).

Thus, we validated four acquired resistance mechanisms, i.e. loss of the drug target, increased expression of multidrug resistance pumps, enhanced activity of the ligand independent signaling pathway and the occurrence of resistant CSC in our long-term molecular evolution assays.

### Combination of tamoxifen with salinomycin overcomes tamoxifen resistance

Finally, we investigated whether the combination of tamoxifen and salinomycin is also superior to the respective single treatments in the tamoxifen resistant MCF-7 TAM-R6 cells. We first examined the effect of salinomycin on cell viability of MCF-7 TAM-R6.

**Figure 5:**
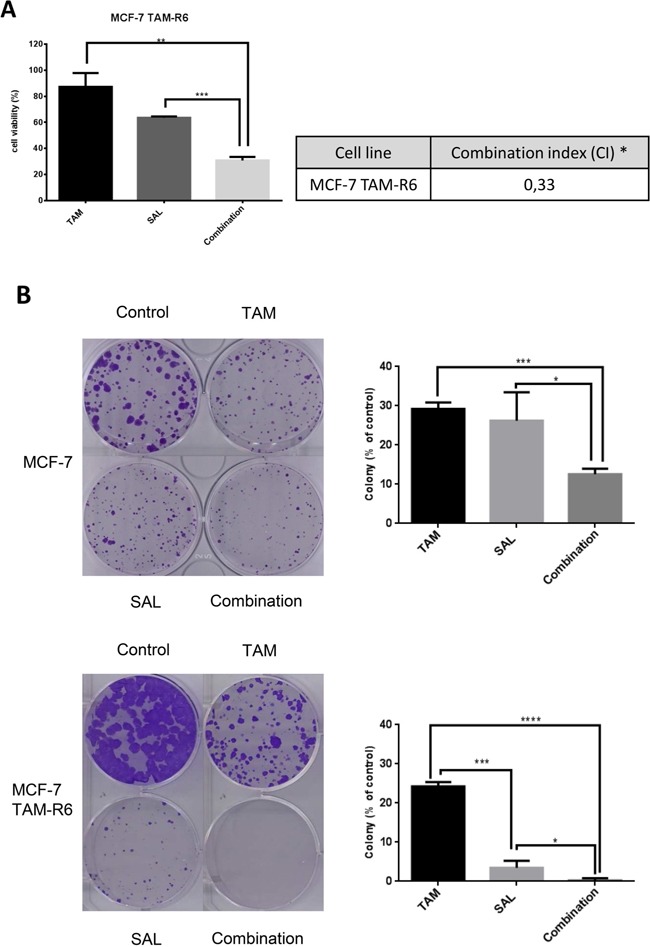
Salinomycin acts synergistic with tamoxifen to circumvent tamoxifen resistance **A.** Combinatorial treatment of tamoxifen resistant cells. Cytotoxicity assay of repeatedly treated MCF-7 cells. MCF-7 TAM-R6 cells were treated with 10μM tamoxifen, 0,5μM salinomycin or their combination for 72h. Subsequently cell viability was assessed by Cell Titer Glo® Assay. N=3; * CI<1: synergistic, CI=1: additive, CI>1 antagonistic. **B.** Clonogenic assay – long-term resistance. To analyze long-term resistance, cells were seeded and incubated for 24h prior to 72h of treatment with 20μM tamoxifen, 10μM salinomycin or their combination. Cells were then grown for 14 days, fixed and stained. Surviving colonies were analyzed by ColonyArea (Image J plug-in), N=3 p-values: *0,05; **0,01; ***0,001; ****0,0001.

Remarkably, tamoxifen resistant cells MCF-7 TAM-R6 are four times more sensitive to salinomycin than parental MCF-7 cells according to their IC_50_ values (Supplementary Figure S3). The combination of both drugs is more efficient in resistant cells than in sensitive ones and shows moreover a synergistic effect [22–24] (Figure 5A). As resistance of tumor cells to a certain drug is not only reflected in short term cell survival, a long-term assay represents efficacy of drugs in another way. Thus, we performed a clonogenic assay where cells could recover for two weeks after the initial 72h drug treatment. We observed an enhanced colony formation in tamoxifen resistant cells, reflecting increased proliferation. In both cell lines, i.e. MCF-7 and MCF-7 TAM-R6, the combination of the drugs shows highest efficacy (Figure 5B).

In summary, we have shown that salinomycin co-treatment is able to overcome tamoxifen resistance in MCF-7 TAM-R6 cells.

## DISCUSSION

Recent findings revealed that salinomycin, a well-known monocarboxylic polyether antibiotic, might be a promising drug in cancer therapy because it is able to eradicate cancer stem cells [11] and to overcome ABC-transporter mediated resistance due to its interference with cell membranes [10]. To figure out whether salinomycin is a suitable additive drug in tamoxifen treatment, it had to be elucidated whether salinomycin co-treatment provides increased antitumoral efficacy. Here, we show that the combinational treatment augments cytotoxicity synergistically and reduces the expression of all Egfr-family members. It was further detected that Egfr as well as Her2 accumulated in lysosomes upon single salinomycin treatment though we observed no significant difference in protein levels but decreased mRNA levels. These facts might reflect enhanced *de novo* protein biosynthesis of the RTKs, compensating the lysosomal degradation induced by salinomycin.

By additionally applying tamoxifen, the ERα-signaling is inhibited leading to the induction of an escape mechanism, i.e. the ligand independent activation of ERα via the Egfr-family. Subsequently, RTK signaling has to become more active reflecting a stimulus that might lead to enhanced receptor accumulation in endosomes.

**Figure 6:**
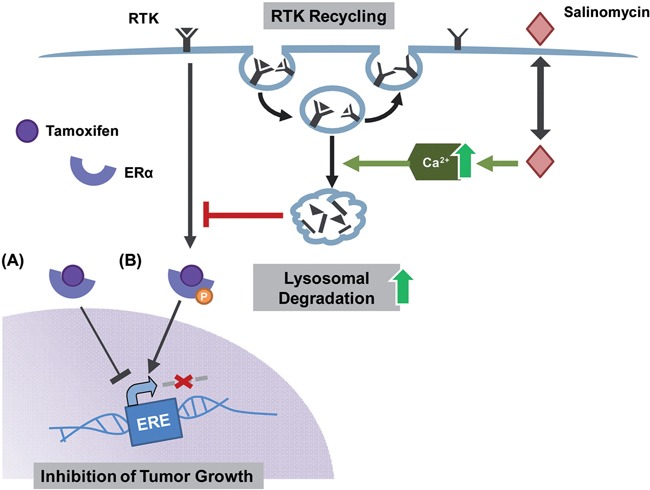
Hypothesis how salinomycin hampers the ligand independent activation of ERα Tamoxifen binds the ERα and subsequently inhibits the transcription of estrogen responsive genes responsible for tumor growth **(A)** Luminal A tumors however are able to circumvent this blockage via the ligand independent phosphorylation of the ERα by enhanced RTK signaling **(B)** A combinatorial treatment of tamoxifen-sensitive as well as -resistant cells with tamoxifen and salinomycin hampers both ways of ERα activation as salinomycin enhances lysosomal degradation of RTK via an increase of Ca^2+^.

Recently, Boehmerle and Endres have demonstrated increased cytosolic Ca^2+^-levels in neuronal cells upon salinomycin treatment [25]. This was ascribed partly to Ca^2+^-efflux from mitochondria and partly to increased Ca^2+^ influx via the plasmamembrane by Na^+^/Ca^2+^ exchangers. These increased cytosolic Ca^2+^-levels induced by salinomycin treatment, facilitate premature fusion of endosomes and lysosomes [25]. Consequently, lysosomal degradation of RTKs could be enhanced causing decreased protein levels (Figure 6). To which extent the general trafficking and recycling of membrane receptors is affected by salinomycin has to be elucidated in future studies.

When specifically hampering the ligand independent pathway by lapatinib, a Egfr- and Her2-inhibitor, we observed a beneficial effect of the combination as well (data not shown). However, combining tamoxifen and salinomycin displayed even greater efficacy. Thus, the combination is more potent due to additional toxic effects of salinomycin like enhanced Ca^2+^-influx or interference with the plasma membrane.

Even though most ERα positive breast tumors initially respond to endocrine therapy, a majority relapses after several years and turns insensitive to tamoxifen treatment [28]. Drug resistance mechanisms can be distinguished between long-term resistance (acquired), which we observe after several treatment cycles, and short-term effects (intrinsic resistance). In this study, we mimicked acquired endocrine resistance by repeated tamoxifen treatment and demonstrated the existence of three major resistance mechanisms, namely, MDRs, CSC and the loss of its target, i.e. ERα. Intrinsic drug resistance was studied by 72 h of tamoxifen treatment and revealed the ligand independent signaling by RTKs. By inhibiting ERα, SERMs induce an escape mechanism via a cross-talk with Egfr-family members [7-9, 29-34]. Salinomycin directly interferes with this pathway and thus is complementing the tamoxifen effect. A change in cancer stem cell marker (CSM) expression and the expression of multiple drug resistance (MDR) proteins was not observed for short term treatment.

However, when analyzing long-term resistance upon single tamoxifen treatment by the molecular evolution assay, we observed acquired resistance mechanisms, i.e. increased MDR and CSM (cancer stem cell markers). As published previously, long-term single salinomycin treatment, results in the opposite mechanisms, i.e. reduced CSM and increased MDR [35], showing the possibility of salinomycin to counteract the acquired resistance mechanism of single tamoxifen treatment.

In order to investigate the efficacy of the combination, salinomycin and tamoxifen, we examined the cell viability in tamoxifen-sensitive (MCF-7) as well as tamoxifen-resistant MCF-7 TAM-R6 cells and showed that a combined treatment is beneficial in both cases. This indicates that the combination of tamoxifen with salinomycin not only avoids intrinsic resistance to endocrine therapy but also eradicates acquired resistant breast cancer cells. Thus, we postulate a novel treatment strategy for luminal A breast cancer patients in order to prevent and to overcome tamoxifen resistance.

## MATERIALS AND METHODS

### Cell culture and drug treatment

The breast cancer cell lines MCF-7 and T47D were cultured according to supplier's instructions (ATCC). Cells were treated at a confluence of 80% for 72h with 10μM tamoxifen (Sigma, Germany) and 0,5μM salinomycin (Sigma, Germany) separately or in combination. For this purpose, drugs were diluted in DMSO which also served as control.

### Cell viability assay

To evaluate the toxicity of the two different drugs, a Cell Titer Glo® Luminescent Cell Viability Assay (Promega) was performed. Here, 3000 cells were seeded in a black clear bottom 96-well plate and 24h later treated with the corresponding drug for another 72h. Afterwards the reagent was added to the cells, followed by 10 min of incubation at room temperature. Finally plates were analyzed with a fluorescence detector.

### IC50 calculation

The IC50 value for each drug was achieved by applying the following equation:

$$\text{Y}=\text{100}/(\text{1}+{\text{10}}^{\left(\text{x}-\text{logIC50}\right)})$$

### Combination index

The combination index (CI) of tamoxifen and salinomycin was calculated according to the following equation [23, 24].

$$\text{CI}=\frac{\text{CA,x}}{\text{ICx,A}}+\frac{\text{CB,x}}{\text{ICx,B}}$$

CI: Combination Index

C_A,x_; C_B,x_: concentrations of drug A or B used in combination to achieve x% cell viability

IC_x,A_; IC_x,B_: concentrations of single agents to achieve the same cell viability

CI<1: synergy

CI=1: additivity

CI>1: antagonism

### Western blot

For immune blotting antibodies Egfr (#2232), Her4 (#4795), p-AKT (#9271), p-ERK1,2 (#9101), GAPDH (#2118) and PARP (#9542) from Cell Signaling Technology^®^ were utilized.

Antibodies against Her2 (#06-562) and Her3 (#05-471) were bought from Merck Millipore, ERα (#7207), AKT (#8312), ERK1,2 (#93) from Santa Cruz Biotechnology^®^, Inc. and α-Tubulin (#T9026) from Sigma-Aldrich®.

Cells were treated for 72h and lyzed with RIPA-buffer containing 1% Triton X. 30μg protein were separated using a SDS-PAGE and afterwards transferred to a nitrocellulose membrane. After one hour blocking with NET-gelatine containing 3% BSA, the blots were incubated overnight at 4°C in the corresponding primary antibody in NET-gelatine, followed by several washing steps with NET-gelatine. Subsequently membranes were incubated for one hour in horseradish peroxidase conjugated anti-mouse or anti-rabbit secondary antibodies at room temperature. After additional washing steps, detection was performed using enhanced chemiluminscence (ECL, GE Healthcare) on X-ray films.

### RNA isolation, cDNA synthesis and qPCR

Total RNA was extracted and isolated from cells using miRCURY™RNA Isolation Kit (Exiqon, Denmark). RNA was then reverse transcribed to cDNA from 1μg of total RNA by using Transcriptor First Strand cDNA Synthesis Kit (Roche). Real time-PCR was then performed in Light Cycler®480 (Roche) using Master Probes Kit (Roche) and Universal Probe Library (UPL) (Roche). All protocols were performed according to the manufacturer's instructions.

RT-PCR was performed using the following primers and UPLs: ER alpha, UPL probe #17, left primer: ATCCACCTGATGGCCAAG, right primer: GCTCCATGCCTTTGTTACTCA; SOX-2, UPL probe #35, left primer: TTGCTGCCTCT TTAAGACTAGGA, right primer: CTGGGGCTCAAACT TCTCTC; Oct-4, UPL probe #35, left primer: AG CAAAACCCGGAGGAGT, right primer: CCACATCG GCCTGTGTATATC; MDR1, UPL probe #7, left primer: CAAGCATCTGCCAAAACCTC, right primer: CTGGGTTTCCCCCTGTAAAT; BCRP1, UPL probe #56, left primer: TGGCTTAGACTCAAGCACAGC, right primer: TCGTCCCTGCTTAGACATCC, Nanog, UPL probe #69, left primer: ATGCCTCACACGGAGACTGT, right primer: AGGGCTGTCCTGAATAAGCA; GAPDH, UPL probe #45, left primer: TCCACTGGCGTCTTCACC, right primer: GGCAGAGATGATGACCCTTTT. GAPDH was used as internal control. The results were analyzed using comparative threshold cycle (ΔΔCT method).

### ERα reporter assay

Cells were transfected with 3x-ERE-TATA-Luc plasmid (#11354 Add gene) [36] using K2® Transfection System (Biontex Laboratories GmbH), according to manufacturer's protocol.

### Spinning disk confocal microscopy

For all imaging experiments cells were seeded in 8 well chambered μ-Slides (ibiTreat, Ibidi GmbH) at a density of 7000 (3 days before imaging) or 10000 (2 days before imaging) cells per well.

To analyze calcium concentration and lysosomes MCF-7 cells were pre-treated with 6μM salinomycin for 3h or 24h or kept without salinomycin treatment. 1 hour before imaging, 6μM Fluo-3-AM and 15nM Lysotracker deep red (both Molecular Probes, Life Technologies) were added to the cells. For imaging, medium was exchanged by marker-free CO_2_ independent medium (with or without salinomycin).

### Egfr and Her2 trafficking

24 hours after seeding, cells were transfected with Egfr eGFP (Addgene plasmid 32751 [37]) or Her2 eGFP plasmid (Addgene Plasmid 39321 [38]) using the X-tremeGENE 9 DNA Transfection Reagent (Roche). Briefly, 1μg DNA were mixed at 1:3 ratio with transfection reagent in 200μl serum free medium and incubated for 20 minutes at room temperature. Subsequently, 5μl of the transfection mix (= 25ng DNA) was pipetted to each well of the cell culture chamber.

Receptor GFP expressing cells were imaged 48-72 hours after transfection. To detect the effect of salinomycin on receptor trafficking, salinomycin was added at 6μM concentration 3h and 24h before imaging.

For co-localization with lysosomes, 15nM lysotracker deep red was incubated for 1 hour on the cells, followed by medium exchange. To induce receptor endocytosis, 50 pmol/ml EGF was added to the cells at 24 hours before measurement.

Spinning disk confocal microscopy was performed on a setup based on the TE200E microscope and the Yokogawa spinning disk unit CSU10. The system was equipped with a Nikon 1.49 NA 100x Plan Apo oil immersion objective. For two color detection, cells were excited with alternating 488nm and 641nm laser light for 270ms and 300ms per frame. Fluorescence was split into two emission channels by a dichroic mirror (565, DCXR chroma) and passed through filter sets (525/50 and 730/140, semrock). Confocal z-stacks of cells were imaged with a spacing of 166nm between two planes. Image sequences of both channels were captured on two EMCCD cameras (iXon; Andor). Images were geometrically calibrated according to reference images with Tetraspeck Beads. Overlay images and LUT images were built in ImageJ.

### Quantification

To quantify the Fluo-3-AM and Lysotracker fluorescence in the cells, digital image analysis was performed in ImageJ: A threshold value for background fluorescence was set according to control images of untreated cells. The number and grey values of pixels above the set threshold was then determined for each image and the integrated intensity was calculated.

(integrated intensity = number of selected pixels * mean grey value of selected pixels). Mean values of all evaluated cells are presented (N=8-10 images for each incubation time).

For co-localization analysis the ImageJ macro JaCOP was applied. According to a manual threshold, the fluorescence signal in the red and green was selected. Next the fraction of green pixels overlapping red pixels was calculated (Manders coefficient, M1). Mean values of all evaluated cells (N=7-13 images) are presented together with the standard error of the mean (SEM).

### Molecular evolution assay

This experiment was performed as described previously [39]. Briefly, MCF-7 cells were treated with 10μM tamoxifen or 0,05μM salinomycin for 72h followed by a recovery phase until a confluence of 80%. Afterwards, cells underwent five further treatment cycles and were harvested for subsequent experiments.

### Rhodamine efflux assay

This assay was performed to measure the pump activity of expressed ABC-transporter. Therefore, cells were trypsinized and diluted to an end concentration of 10^6^ cells/ml with growth media.

Afterwards cells were treated with 0,2 μg/ml rhodamine for 30 min, centrifuged and resuspended in 1ml media. Finally, the samples were analyzed by flow cytometry after the indicated time points.

### Mammosphere forming assay

To figure out the cancer stem cell properties of cell populations, we utilized the mammosphere forming assay. For this test 10 000 cells/well were seeded in ultra-low adherent plates (Corning) and cultivated in conditioned media for at 7 days. Afterwards spheroids were counted.

### Clonogenic assay

To perform the clonogenic assay, 10^3^ cells were seeded in 6-well-plates (TPP; Switzerland), cultured for 24h and subsequently treated for 72h with tamoxifen, salinomycin and the combination.

Cells were then grown for 14 days, fixed and stained with paraformaldehyde (PFA) containing crystal violet. Surviving colonies were analyzed by ColonyArea, as previously described [40].

## SUPPLEMENTARY MATERIALS FIGURES


