# Supplementary material for: Salinomycin co-treatment enhances tamoxifen cytotoxicity in luminal A breast tumor cells by facilitating lysosomal degradation of receptor tyrosine kinases
Source: Oncotarget. 2016 Jul 7;7(31):50461–76. doi: 10.18632/oncotarget.10459 (PMC5226596; doi:10.18632/oncotarget.10459)
Supplement: Supplementary file 1 [file oncotarget-07-50461-s001.pdf]

Salinomycin co-treatment enhances tamoxifen cytotoxicity in luminal A breast tumor cells by facilitating lysosomal degradation of receptor tyrosine kinases

SUPPLEMENTARY MATERIALS

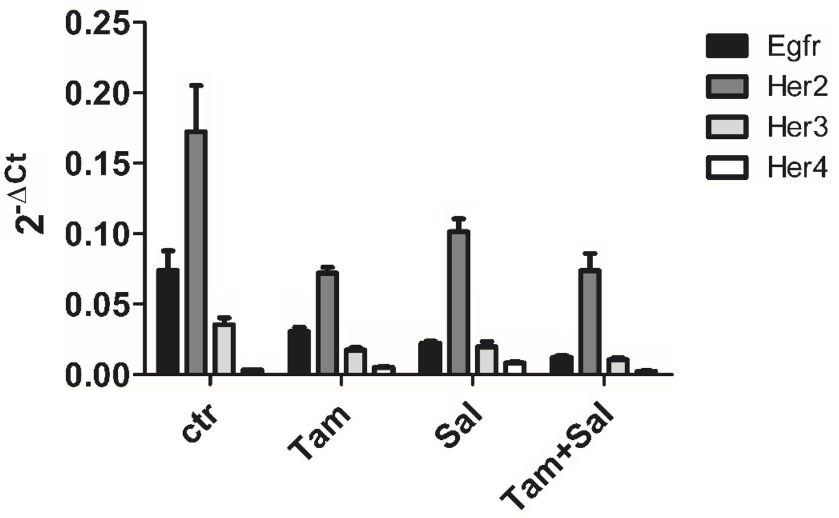

Supplementary Figure S1: qPCR of Egfr-family members in MCF-7 after 72h treatment with 10µM tamoxifen, 0,5µM salinomycin or the combination thereof.

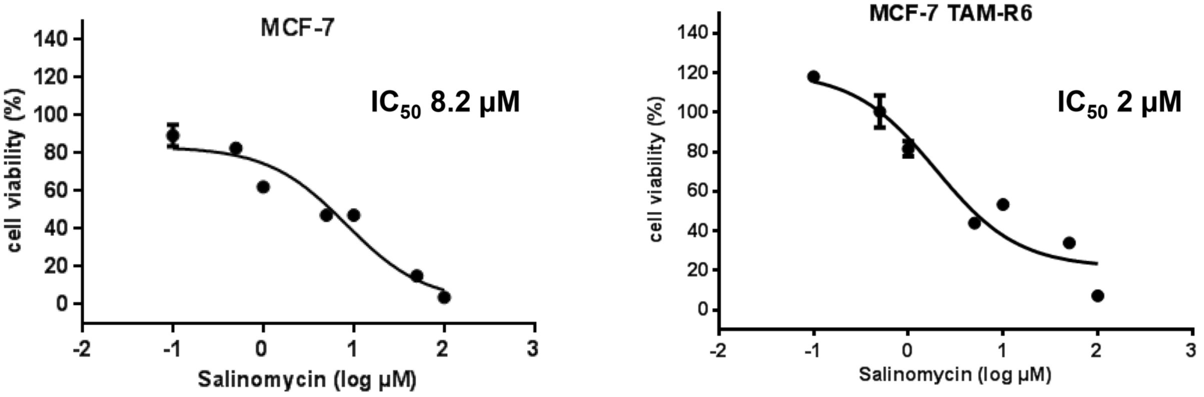

Supplementary Figure S2: Time projection of HeLa cells treated with 6µM of salinomycin and analyzed by spinning disc microscopy.

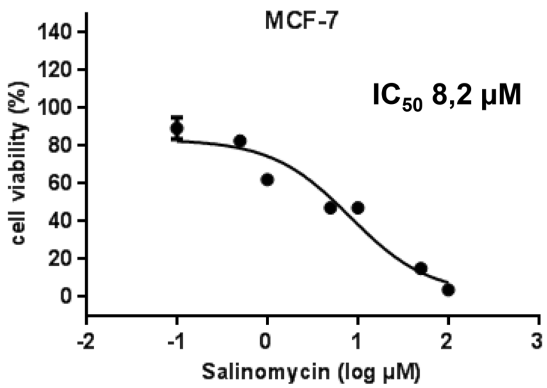

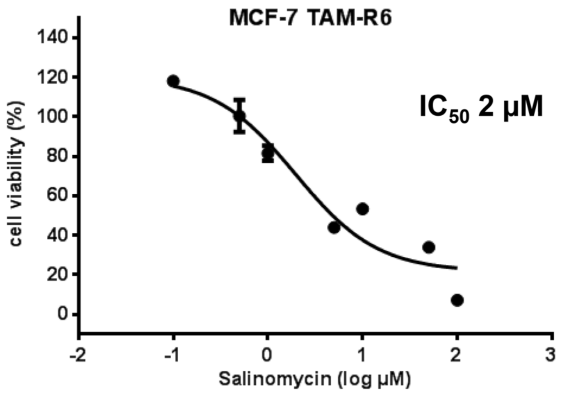

Supplementary Figure S3: IC_{50} of salinomycin in MCF-7 TAM-R6. Tamoxifen resistant cells were treated with indicated concentrations of salinomycin for 72h and afterwards analyzed by Cell Titer Glo® Assay.

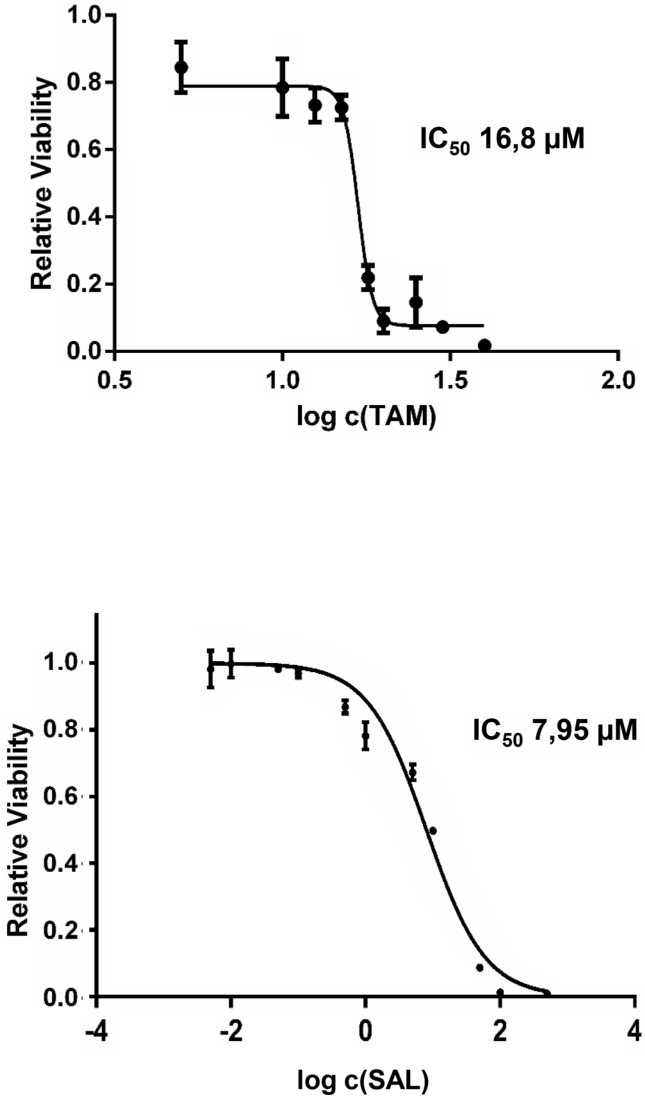

Supplementary Figure S4: IC_{50} of tamoxifen and salinomycin in T47D. T47D were treated with indicated concentrations of tamoxifen or salinomycin for 72h and analyzed by Cell Titer Glo® Assay.
